# Intrinsic Frequency Analysis and Fast Algorithms

**DOI:** 10.1038/s41598-018-22907-4

**Published:** 2018-03-20

**Authors:** Peyman Tavallali, Hana Koorehdavoudi, Joanna Krupa

**Affiliations:** 1 Division of Engineering and Applied Sciences, California Institute of Technology, 1200 East California Boulevard, MC 205-45, Pasadena, CA 91125 USA; 2 Aerospace and Mechanical Engineering, University of Southern California, Los Angeles, CA 90089-1453 USA; 3 Avicena LLC, 2400 N Lincoln Ave, Altadena, CA 91001 USA

## Abstract

Intrinsic Frequency (IF) has recently been introduced as an ample signal processing method for analyzing carotid and aortic pulse pressure tracings. The IF method has also been introduced as an effective approach for the analysis of cardiovascular system dynamics. The physiological significance, convergence and accuracy of the IF algorithm has been established in prior works. In this paper, we show that the IF method could be derived by appropriate mathematical approximations from the Navier-Stokes and elasticity equations. We further introduce a fast algorithm for the IF method based on the mathematical analysis of this method. In particular, we demonstrate that the IF algorithm can be made faster, by a factor or more than 100 times, using a proper set of initial guesses based on the topology of the problem, fast analytical solution at each point iteration, and substituting the brute force algorithm with a pattern search method. Statistically, we observe that the algorithm presented in this article complies well with its brute-force counterpart. Furthermore, we will show that on a real dataset, the fast IF method can draw correlations between the extracted intrinsic frequency features and the infusion of certain drugs.

## Introduction

Cardiovascular diseases (CVDs) relate to different conditions which can affect the performance of the heart and blood vessels. These diseases include: coronary artery disease, valvular heart disease, cardiomyopathy, heart rhythm disturbances and heart infections. As an example, congestive heart failure is a disease which happens when the heart does not work normally and cannot provide enough blood flow for body tissues. In this disease, the heart muscle does not stretch and contract in a normal way. Another example of CVDs is pulmonary hypertension which is a condition that there is a high blood pressure in the arteries that go from heart to the lungs. In some cases, the arteries in the lung become narrow or blocked. Therefore, blood flow harder through the arteries and this causes blood pressure to further increase in lungs making the blood flow even harder in this organ. All these changes will affect arterial pressure waveforms.

Cardiovascular diseases and stroke are major causes of death in the United States. The total cost related to CVDs and stroke was estimated to be more than $316 billion in 2011–2012^1,2^. The American Heart Association (AHA) estimates that in 2030 the direct costs will reach $818 billion^3^. It is mentioned that the indirect costs for 2030 would be $276 billion^3^. Hence, clinical measurements of cardiovascular health indices are of great importance. These methods and measurements are essential tools for monitoring cardiovascular health due to their relative availability. For example, Left Ventricular Ejection Fraction (LVEF) is a measure of left ventricular contractility^4^. Carotid-Femoral Pulse Wave Velocity (cfPWV) is a measure of aortic stiffness^5^. Velocity-encoded (VENC) phase contrast Magnetic Resonance Imaging (MRI) helps to detect the existence of flow vorticity that measures the morphological changes of the cardiac chamber wall^6^.

However, current methods of measuring such indices are expensive, sometimes invasive, prone to measurement errors, and not necessarily easy to use. For example, 2D LVEF echocardiography is not accurate compared to more expensive and laborious gold standard cardiac MRI method^7–10^. As another example, obtaining accurate cfPWV measurements often requires certain medical devices and a well-trained staff within a clinical setting^11^. Consequently, continuous measurement of these indices is not practical. These limitations emphasize the need for new cardiovascular monitoring methods.

Intrinsic Frequency (IF) has been established as a new method of cardiovascular monitoring through a novel signal processing methodology^12^. The IF method needs only an uncalibrated pulse pressure^13^ to extract pertinent information regarding the cardiovascular health of an individual^12^. The IF method has also been shown to be capable of non-invasively measuring LVEF by means of an iPhone camera^14^. We believe that methods like IF are of clinical and financial benefit in addressing cardiovascular monitoring.

In this paper, at first, we provide an overview of the IF method. Next, we present an approximate derivation of the IF model by combining Navier-Stokes equations and continuity with elasticity equations. This helps to build a solid mathematical foundation for the IF method and the analysis that follows. Later, we analyze the IF algorithm in the space of feasible solutions, and based on that, we introduce a new version of the IF algorithm which is faster than the current brute-force IF method^13^ while maintaining the same accuracy. We then perform a case study on real pressure waveforms drawn from canine data using our new algorithm. We will see that the fast IF algorithm is capable of capturing the effects of different drug infusions on a canine subject.

## Brief Overview of IF Method

### A History of Analyzing Cardiovascular Pulse Waveform

Blood pressure was first measured by Hales in 1735^15^. In his measurements, he found that blood pressure is not constant in the arterial system. He related these variations to the elasticity of the arteries^15^. Currently, it is known that the shape of the arterial pulse wave is intimately related to the physiology and pathology of the whole arterial system^16^. There has been much research on analyzing the dynamics of blood pressure and flow in arterial systems^17–21^. Specifically, there are two main approaches to analyzing cardiovascular pulse wave data. One approach is based on a systematic mathematical framework for the cardiovascular system. The other is based on directly analyzing the pulse pressure waveform using signal processing methods.

An example of the systematic framework can be seen with the set of Windkessel models^22^. The formulation of a minimal lumped model of the arterial system was first presented by Westerhof *et al*.^22^. Based on a Windkessel model, the arterial system dynamics have been modeled through a combination of different elements such as resistance, compliance and impedance. In this simplified model of the arterial system, the blood flow dynamics is modeled by the interaction between the elements (assuming the blood flow acts as the current in the system). Because of the type of modeling, the wave transmission of the blood flow is neglected. As a result, the Windkessel models is not able to represent the entire dynamics of the blood flow in an arterial system accurately.

On the other hand, there are various methods for direct analysis of an arterial pulse waveform, in both time and frequency domains^20^. For example, the impedance method, which is based on Fourier transform, is a common method to analyze the pressure waveform in the frequency domain^17^. As an example, Milnor has shown that the pressure and flow waveforms can be a superposition of several harmonics using the Fourier method^23^. Another method to investigate the pressure wave in the time domain is the wave intensity analysis which is based on wavelet transform^24^. These methods do not necessarily convey a physical understanding of the cardiovascular system.

The IF algorithm presented in^13^ is analyzing a pulse waveform through a direct time-frequency signal processing machinery setting, from a quantitative perspective. Although, in previous work^12^, we tried to qualitatively express a systems approach to the IF formulation, the quantitative picture has not yet been clear. However, in this article, we show this connection from a quantitative perspective.

### IF Formulation

In the IF method, the aortic pressure waveform at time $t\in [0,T)$, for a cardiac period *T*, can be represented as

2.1 $$\begin{array}{rcl}S({a}_{i},{b}_{i},\bar{p},{\omega }_{i};t) & = & ({a}_{1}\,\cos \,{\omega }_{1}t+{b}_{1}\,\sin \,{\omega }_{1}t+\bar{p})\,{{\bf{1}}}_{[\mathrm{0,}{T}_{0})}\,(t)\\  &  & +\,({a}_{2}\,\cos \,{\omega }_{2}t+{b}_{2}\,\sin \,{\omega }_{2}t+\bar{p})\,{{\bf{1}}}_{[{T}_{0},T)}\,(t),\end{array}$$

with a continuity condition at *T*_0_ and periodicity at *T*. In this formulation, the *indicator function* is defined as

$${{\bf{1}}}_{[x,y)}\,(t)=\{\begin{array}{cc}1, & x\le t < y,\\ 0, & else\mathrm{.}\end{array}$$

Here, *a*_1_, *b*_1_, *a*_2_ and *b*_2_ are the envelopes of the IF model fit. *ω*_1_ and *ω*_2_ are the Intrinsic Frequencies (IFs) of the waveform. Further, $\bar{p}$ is the mean pressure during the cardiac cycle. This type of formulation embeds the coupling and decoupling of heart and aorta.

The goal of the IF model (2.1) is to extract a fit, called Intrinsic Mode Function (IMF), that carries most of the energy (information) from an observed pressure waveform *f*(*t*) in one period. The latter is done by solving the following optimization problem^13^:

22 $$\mathop{minimize}\limits_{{a}_{i},{b}_{i},{\omega }_{i},\bar{p}}\quad \parallel f(t)-S({a}_{i},{b}_{i},\bar{p},{\omega }_{i};t){\parallel }_{2}^{2}$$

*subject to*

2.3 $$\begin{array}{rcl}{a}_{1}\,\cos \,{\omega }_{1}{T}_{0}+{b}_{1}\,\sin \,{\omega }_{1}{T}_{0} & = & {a}_{2}\,\cos \,{\omega }_{2}{T}_{0}+{b}_{2}\,\sin \,{\omega }_{2}{T}_{0},\\ {a}_{1} & = & {a}_{2}\,\cos \,{\omega }_{2}T+{b}_{2}\,\sin \,{\omega }_{2}T\mathrm{.}\end{array}$$

Here, $\parallel {\parallel }_{2}$ is the *L*^2^-norm. The first linear condition in this optimization enforces the continuity of the extracted IMF at the dicrotic notch. The second one imposes the periodicity. The mathematical convergence and accuracy of the IF algorithm have been explained in a previous work^13^. In the next sections, we explore the foundation of the IF algorithm and propose a faster IF algorithm.

## Approximate Derivation of the IF Model

As mentioned earlier, in a previous work^12^, we tried to express a systems approach to the IF formulation qualitatively. However, in this article, we show this connection from a quantitative perspective. This section is devoted to this purpose.

Here we use a simplified model to address our approach. However, for a more general modeling, analysis and estimation of the blood flow and pressure estimation one could see^25,26^.

**Figure 1:**
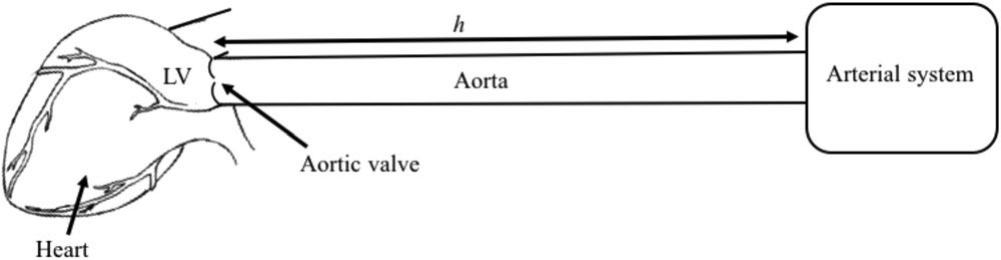
Simplified cardiovascular system model schematic.

In this paper, we assume that the Left Ventricle (LV), the aortic valve, aorta and the arterial system can be represented by a simplified model as shown in Fig. 1. Here, the LV and the aortic valve are assumed to be the boundary condition at the entrance of the aortic tube and the arterial system is the terminal boundary condition of the aortic tube. The boundary condition at the entrance of the aortic tube changes from an LV boundary condition to a closed valve boundary condition, at the dicrotic notch time *T*_0_ during a cardiac cycle [0, *T*]. We further assume that blood is a Newtonian incompressible fluid, the aorta is a straight and sufficiently long elastic tube with a constant circular cross section and there is no external force causing flow rotation. These assumptions are not all satisfied in a real cardiovascular system. However, they are useful in estimating the general behavior of blood in aorta.

Combining the Navier-Stokes equations and continuity with the elasticity equation, we can drive a model for the flow *Q*(*x*, *t*) and the pressure *P*(*x*, *t*) along the length *x* of an aorta as follow

31 $$-\frac{\partial P}{\partial x}\,(x,t)=L\frac{\partial Q}{\partial t}\,(x,t)+RQ\,(x,t),$$

32 $$-\frac{\partial Q}{\partial x}\,(x,t)=C\frac{\partial P}{\partial t}\,(x,t).$$

The step by step derivation of these equations is presented in the supplementary material. Parameters *L*, *R*, and *C* represent inductance, resistance, and compliance of the blood in aorta. Here, 0 ≤ *x* ≤ *h*, where *h* represents the aortic length. This model has also been discussed and simulated numerically in^27^ with a complex set of boundary conditions. Here, our main concentration will be on the aortic tube oscillatory waveform solutions. Next, we will show that we can derive (2.1) from (3.1) and (3.2).

Since the input to the IF model (2.1) is a pressure waveform, we need to extract an equation for the pressure *P*(*x*, *t*) from Equations (3.1) and (3.2) by eliminating the flow. Combining Equations (3.1) and (3.2) results in

3.3 $$CL\frac{{\partial }^{2}P}{\partial {t}^{2}}\,(x,t)+CR\frac{\partial P}{\partial t}\,(x,t)=\frac{{\partial }^{2}P}{\partial {x}^{2}}\,(x,t).$$

A similar expression could also be found for the flow field *Q*(*x*, *t*). Taking $P(x,t)={\mathscr{K}}(t)p(x,t)+\bar{p}$, with $\bar{p}$ as the the mean pressure, we can write equation (3.3) as

3.4 $$\begin{array}{l}(CL\ddot{{\mathscr{K}}}\,(t)+CR\dot{{\mathscr{K}}}\,(t))p(x,t)+(2CL\dot{{\mathscr{K}}}\,(t)+CR{\mathscr{K}}\,(t))\,\frac{\partial p}{\partial t}\,(x,t)+CL{\mathscr{K}}\,(t)\frac{{\partial }^{2}p}{\partial {t}^{2}}\,(x,t)\\ \quad =\,{\mathscr{K}}(t)\frac{{\partial }^{2}p}{\partial {x}^{2}}\,(x,t).\end{array}$$

Here, we have used the dot notation to represent the time derivative. We can simplify the term in front of $\frac{\partial p}{\partial t}(x,t)$, in (3.4), by setting $2C\,L\dot{{\mathscr{K}}}(t)+C\,R{\mathscr{K}}(t)=0$. The latter has a solution ${\mathscr{K}}(t)=K{e}^{-\frac{R}{2L}t}$ for some constant *K*. This reduces Equation (3.4) into

3.5 $$CL\frac{{\partial }^{2}p}{\partial {t}^{2}}\,(x,t)-\frac{C{R}^{2}}{4L}p\,(x,t)=\frac{{\partial }^{2}p}{\partial {x}^{2}}\,(x,t).$$

The solution of Equation (3.5) can be expressed in terms of eigenfunctions. In other words, using the method of separation of the variables, one can express the solution of Equation (3.5) as

3.6 $$p\,(x,t)=\sum _{n=1}^{\infty }\,{T}_{n}(t){X}_{n}\,(x)$$

for

3.7 $${T}_{n}\,(t)={\alpha }_{n}\,sin\,({\omega }_{n}t)+{\beta }_{n}\,cos\,({\omega }_{n}t),$$

3.8 $${X}_{n}\,(x)={\zeta }_{n}\,sin\,(\sqrt{CL\,{({\omega }_{n})}^{2}-\frac{C{R}^{2}}{4L}}x)+{\eta }_{n}\,cos\,(\sqrt{CL\,{({\omega }_{n})}^{2}-\frac{C{R}^{2}}{4L}}x),$$

and some constants *α*_*n*_, *β*_*n*_, *ζ*_*n*_ and *η*_*n*_. As a result, the solution of (3.3) can be expressed as

39 $$\begin{array}{rcl}P\,(x,t) & = & \bar{p}+K{e}^{-\frac{R}{2L}t}\,\sum _{n=1}^{\infty }\,\{({\alpha }_{n}\,sin\,({\omega }_{n}t)+{\beta }_{n}\,cos\,({\omega }_{n}t))\\  &  & \times \,({\zeta }_{n}\,sin\,(\sqrt{CL\,{({\omega }_{n})}^{2}-\frac{C{R}^{2}}{4L}}x)+{\eta }_{n}\,cos\,(\sqrt{CL\,{({\omega }_{n})}^{2}-\frac{C{R}^{2}}{4L}}x))\}.\end{array}$$

The variables *ω*_*n*_ can be expressed based on the boundary conditions of the aortic tube. We need to emphasize that for a period of the cardiac cycle [0, *T*), the boundary conditions change before and after the dicrotic notch *T*_0_. Hence, for $t\in [0,T)$, Equation (3.9) can be written as

310 $$\begin{array}{rcl}P\,(x,t) & = & \bar{p}+{{\bf{1}}}_{[\mathrm{0,}{T}_{0})}\,(t)\,{K}^{1}{e}^{-\frac{R}{2L}t}\,\sum _{n=1}^{\infty }\{({\alpha }_{n}^{1}\,sin\,({\omega }_{n}^{1}t)+{\beta }_{n}^{1}\,cos\,({\omega }_{n}^{1}t))\\  &  & \times \,({\zeta }_{n}^{1}\,sin\,(\sqrt{CL\,{({\omega }_{n}^{1})}^{2}-\frac{C{R}^{2}}{4L}}x)+{\eta }_{n}^{1}\,cos\,(\sqrt{CL\,{({\omega }_{n}^{1})}^{2}-\frac{C{R}^{2}}{4L}}x))\}\\  &  & +\,{{\bf{1}}}_{[{T}_{0},T)}\,(t)\,{K}^{2}{e}^{-\frac{R}{2L}t}\,\sum _{n=1}^{\infty }\,\{({\alpha }_{n}^{2}\,sin\,({\omega }_{n}^{2}t)+{\beta }_{n}^{2}\,cos\,({\omega }_{n}^{2}t))\\  &  & \times \,({\zeta }_{n}^{2}\,sin\,(\sqrt{CL\,{({\omega }_{n}^{2})}^{2}-\frac{C{R}^{2}}{4L}}x)+{\eta }_{n}^{2}\,cos\,(\sqrt{CL\,{({\omega }_{n}^{2})}^{2}-\frac{C{R}^{2}}{4L}}x))\}.\end{array}$$

Here, the superscripts indicated with “1” belong to the form of the solution before the closure of the aortic valve, and the superscripts indicated with “2” belong to the form of the solution after the closure of the aortic valve. Constants *K*^1^, ${\alpha }_{n}^{1}$, ${\beta }_{n}^{1}$, ${\zeta }_{n}^{1}$, ${\eta }_{n}^{1}$ and ${\omega }_{n}^{1}$ are found from the boundary and initial conditions at systole. Similarly, constants *K*^2^, ${\alpha }_{n}^{2}$, ${\beta }_{n}^{2}$, ${\zeta }_{n}^{2}$, ${\eta }_{n}^{2}$ and ${\omega }_{n}^{2}$ are found from the boundary and initial conditions at diastole.

Equation (3.10) is explicitly showing the coupling and decoupling of heart and aorta before and after the dicrotic notch. As the boundary conditions change during a cardiac cycle, the frequencies of oscillation also change from ${\omega }_{n}^{1}$ to ${\omega }_{n}^{2}$. Generally, Equation (3.9) can represent pressure waveform for a Newtonian incompressible fluid in a straight and sufficiently long elastic tube with constant circular cross section.

If the pressure is recorded at a specific point *x*_0_ on aorta, the terms containing the spacial variable *x* would be fixed. In other words, Equation (3.10) would reduce to

311 $$\begin{array}{rcl}P\,(x={x}_{0},t) & = & \bar{p}+\,\{{K}^{1}{e}^{-\frac{R}{2L}t}\,\sum _{n=1}^{\infty }\,{\kappa }_{n}^{1}\,({\alpha }_{n}^{1}\,sin\,({\omega }_{n}^{1}t)+{\beta }_{n}^{1}\,cos\,({\omega }_{n}^{1}t))\}\,{{\bf{1}}}_{[0,{T}_{0})}\,(t)\\  &  & +\,\{{K}^{2}{e}^{-\frac{R}{2L}t}\,\sum _{n=1}^{\infty }\,{\kappa }_{n}^{2}\,({\alpha }_{n}^{2}\,sin\,({\omega }_{n}^{2}t)+{\beta }_{n}^{2}\,cos\,({\omega }_{n}^{2}t))\}\,{{\bf{1}}}_{[{T}_{0},T)}\,(t),\end{array}$$

for

$${\kappa }_{n}^{1}={\zeta }_{n}^{1}\,sin\,(\sqrt{CL\,{({\omega }_{n}^{1})}^{2}-\frac{C{R}^{2}}{4L}}{x}_{0})+{\eta }_{n}^{1}\,cos\,(\sqrt{CL\,{({\omega }_{n}^{1})}^{2}-\frac{C{R}^{2}}{4L}}{x}_{0})$$

and

$${\kappa }_{n}^{2}={\zeta }_{n}^{2}\,sin\,(\sqrt{CL\,{({\omega }_{n}^{2})}^{2}-\frac{C{R}^{2}}{4L}}{x}_{0})+{\eta }_{n}^{2}\,cos\,(\sqrt{CL\,{({\omega }_{n}^{2})}^{2}-\frac{C{R}^{2}}{4L}}{x}_{0}).$$

Now, considering that the cardiac cycle length would be around 1.5 *sec*, at most, and taking into account that *R* is smaller than *L*^27^, one can use the approximation ${e}^{-\frac{R}{2L}t}\simeq 1$. Hence, Equation (3.11) would become

3.12 $$\begin{array}{rcl}P\,(x={x}_{0},t) & \approx  & \bar{p}+\,\{{K}^{1}\,\sum _{n=1}^{\infty }\,{\kappa }_{n}^{1}\,({\alpha }_{n}^{1}\,sin\,({\omega }_{n}^{1}t)+{\beta }_{n}^{1}\,cos\,({\omega }_{n}^{1}t))\}\,{{\bf{1}}}_{[0,{T}_{0})}\,(t)\,\\  &  & +\,\{{K}^{2}\,\sum _{n=1}^{\infty }\,{\kappa }_{n}^{2}\,(({\alpha }_{n}^{2}\,sin\,({\omega }_{n}^{2}t)+{\beta }_{n}^{2}\,cos\,({\omega }_{n}^{2}t))\}\,{{\bf{1}}}_{[{T}_{0},T)}\,(t)\mathrm{.}\end{array}$$

Further, if most of the information, or energy, is carried out by the first terms in the series of the solution, we can further write the approximated solution (3.12) as

313 $$\begin{array}{rcl}P\,(x={x}_{0},t) & \approx  & \bar{p}+\,\{{K}^{1}{\kappa }_{1}^{1}\,({\alpha }_{1}^{1}\,sin\,({\omega }_{1}^{1}t)+{\beta }_{1}^{1}\,cos\,({\omega }_{1}^{1}t))\}\,{{\bf{1}}}_{[0,{T}_{0})}\,(t)\,\\  &  & +\,\{{K}^{2}{\kappa }_{1}^{2}\,({\alpha }_{1}^{2}\,sin\,({\omega }_{1}^{2}t)+{\beta }_{1}^{2}\,cos\,({\omega }_{1}^{2}t))\}\,{{\bf{1}}}_{[{T}_{0},T)}\,(t)\mathrm{.}\end{array}$$

Now, by relabeling

3.14 $${b}_{1}={K}^{1}{\kappa }_{1}^{1}{\alpha }_{1}^{1},$$

3.15 $${a}_{1}={K}^{1}{\kappa }_{1}^{1}{\beta }_{1}^{1},$$

3.16 $${b}_{2}={K}^{2}{\kappa }_{1}^{2}{\alpha }_{1}^{2},$$

3.17 $${a}_{2}={K}^{2}{\kappa }_{1}^{2}{\beta }_{1}^{2},$$

3.18 $${\omega }_{1}={\omega }_{1}^{1},$$

3.19 $${\omega }_{2}={\omega }_{1}^{2},$$

we can approximate the IF model (2.1). The continuity and periodicity conditions (2.3) can also be approximated if we hold the assumption that most of the energy is carried out by the first terms in the series of the solution (3.12).

In short, in this section, we have presented an approximate quantitative justification on the origins of the IF method. In the next section, we move on with the analysis of the optimization problem (2.2) subject to (2.3).

## Analysis of The IF Algorithm

Practically, one must solve the discrete version of (2.2). We assume that the pressure waveform *f*(*t*) is sampled uniformly. Also, we can simplify (2.2) by the fact that any sinusoid can be assumed to start from time *t* = 0 with a compensation coming from a phase shift. In other words, any sinusoid can be expressed as *A* cos *ωt* + *B* sin *ωt*, irrespective of whether the initial time is *t* = 0 or *t* = *T*_0_. Hence, the discrete format of (2.2) can be expressed as

41 $$\begin{array}{cc}\mathop{minimize}\limits_{{a}_{i},{b}_{i},{\omega }_{i},\bar{p}} & \parallel {\bf{f}}-{\bf{S}}\,({a}_{i},{b}_{i},\bar{p},{\omega }_{i};{\bf{t}}){\parallel }_{2}^{2}\\ subject\,to & \begin{array}{rcl}{a}_{1}\,\cos \,{\omega }_{1}{T}_{0}+{b}_{1}\,\sin \,{\omega }_{1}{T}_{0} & = & {a}_{2},\\ {a}_{1} & = & {a}_{2}\,\cos \,{\omega }_{2}\,(T-{T}_{0})+{b}_{2}\,\sin \,{\omega }_{2}\,(T-{T}_{0}),\end{array}\end{array}$$

for **f** = (*f*_1_, …, *f*_*n*+*m*_)′ as the uniform sampling of the original cycle. Here, by taking

4.2 $${\bf{t}}=({{\bf{t}}{\boldsymbol{^{\prime} }}}_{1},{{\bf{t}}{\boldsymbol{^{\prime} }}}_{2})^{\prime} =({t}_{1}^{1},{t}_{1}^{2},\ldots ,{t}_{1}^{n},{t}_{2}^{1},{t}_{2}^{2},\ldots ,{t}_{2}^{m})^{\prime} \in {{\mathbb{R}}}^{(n+m)\times 1}$$

for ${{\bf{t}}}_{1}=(0,{\rm{\Delta }}t,2{\rm{\Delta }}t,\ldots ,{T}_{0})^{\prime} \in {{\mathbb{R}}}^{n\times 1}$ and ${{\bf{t}}}_{2}=({\rm{\Delta }}t,2{\rm{\Delta }}t,\ldots ,T-{T}_{0})^{\prime} \in {{\mathbb{R}}}^{m\times 1}$, we have the discrete form of $S({a}_{i},{b}_{i},\bar{p},{\omega }_{i};t)$ as

43 $${\bf{S}}\,({a}_{i},{b}_{i},\bar{p},{\omega }_{i};{\bf{t}})=(\begin{array}{c}{a}_{1}\,\cos \,{\omega }_{1}{{\bf{t}}}_{1}+{b}_{1}\,\sin \,{\omega }_{1}{{\bf{t}}}_{1}\\ {a}_{2}\,\cos \,{\omega }_{2}{{\bf{t}}}_{2}+{b}_{2}\,\sin \,{\omega }_{2}{{\bf{t}}}_{2}\end{array})+\bar{p}{\bf{1}}.$$

In this article, (.)′ denotes the transpose operator and the vector ${\bf{1}}=(1,1,\ldots ,1)^{\prime} \in {{\mathbb{R}}}^{(n+m)\times 1}$. Also,

4.4 $$\begin{array}{rcl}\cos \,{\omega }_{1}{{\bf{t}}}_{1} & = & (\cos \,{\omega }_{1}{t}_{1}^{1},\ldots ,\,\cos \,{\omega }_{1}{t}_{1}^{n})^{\prime} ,\\ \sin \,{\omega }_{1}{{\bf{t}}}_{1} & = & (\sin \,{\omega }_{1}{t}_{1}^{1},\ldots ,\,\sin \,{\omega }_{1}{t}_{1}^{n})^{\prime} ,\\ \cos \,{\omega }_{2}{{\bf{t}}}_{2} & = & (\cos \,{\omega }_{2}{t}_{2}^{1},\ldots ,\,\cos \,{\omega }_{2}{t}_{2}^{m})^{\prime} ,\\ \sin \,{\omega }_{2}{{\bf{t}}}_{2} & = & (\sin \,{\omega }_{2}{t}_{2}^{1},\ldots ,\,\sin \,{\omega }_{2}{t}_{2}^{m})^{\prime} \mathrm{.}\end{array}$$

The constraints, in (4.1), can be written as

45 $$(\begin{array}{cccc}\cos \,{\omega }_{1}{T}_{0} & -1 & \sin \,{\omega }_{1}{T}_{0} & 0\\ 1 & -\cos \,{\omega }_{2}\,(T-{T}_{0}) & 0 & -\sin \,{\omega }_{2}\,(T-{T}_{0})\end{array})\,(\begin{array}{c}{a}_{1}\\ {a}_{2}\\ {b}_{1}\\ {b}_{2}\end{array})=(\begin{array}{c}0\\ 0\end{array}).$$

If we can solve for two, out of four, unknowns in (4.5), we would make (4.1) an unconstrained optimization. However, it is important to check whether the matrix in (4.5) is of full rank or not. In fact, the rows of this matrix are linearly independent except when

4.6 $$\cos \,{\omega }_{1}{T}_{0}\,\cos \,{\omega }_{2}\,(T-{T}_{0})=1.$$

This will lead into two cases:

1. *Degenerate Case* in which Equation (4.6) holds,
2. *General Case* in which, it does not.

### General Case (cos *ω*_1_*T*_0_ cos *ω*_2_(*T* − *T*_0_) ≠ 1)

One can solve the constraints in (4.1) for *a*_1_ and *a*_2_ to obtain

4.7 $${a}_{1}=\frac{{b}_{1}\,\sin \,{\omega }_{1}{T}_{0}\,\cos \,{\omega }_{2}\,(T-{T}_{0})+{b}_{2}\,\sin \,{\omega }_{2}\,(T-{T}_{0})}{1-\,\cos \,{\omega }_{1}{T}_{0}\,\cos \,{\omega }_{2}\,(T-{T}_{0})},$$

4.8 $${a}_{2}=\frac{{b}_{1}\,\sin \,{\omega }_{1}{T}_{0}+{b}_{2}\,\cos \,{\omega }_{1}{T}_{0}\,\sin \,{\omega }_{2}\,(T-{T}_{0})}{1-\,\cos \,{\omega }_{1}{T}_{0}\,\cos \,{\omega }_{2}\,(T-{T}_{0})}.$$

Equations (4.7) and (4.8) would then simplify (4.3) into

4.9 $${\bf{S}}\,({\omega }_{1},{\omega }_{1},{b}_{1},{b}_{2},\bar{p};{\bf{t}})={\bf{Q}}\,({\omega }_{1},{\omega }_{1},{b}_{1},{b}_{2};{\bf{t}})+\bar{p}{\bf{1}},$$

where **Q**(*ω*_1_, *ω*_1_, *b*_1_, *b*_2_; **t**) = *b*_1_**v**_1_(*ω*_1_, *ω*_2_; **t**) + *b*_2_**v**_2_(*ω*_1_, *ω*_2_; **t**) for

410 $${{\bf{v}}}_{1}\,(({\omega }_{1},{\omega }_{2};{\bf{t}}))=(\begin{array}{l}\frac{\sin \,{\omega }_{1}{T}_{0}\,\cos \,{\omega }_{2}\,(T-{T}_{0})}{1-\,\cos \,{\omega }_{1}{T}_{0}\,\cos \,{\omega }_{2}\,(T-{T}_{0})}\,\cos \,{\omega }_{1}{{\bf{t}}}_{1}+\,\sin \,{\omega }_{1}{{\bf{t}}}_{1}\\ \frac{\sin \,{\omega }_{1}{T}_{0}}{1-\,\cos \,{\omega }_{1}{T}_{0}\,\cos \,{\omega }_{2}\,(T-{T}_{0})}\,\cos \,{\omega }_{2}{{\bf{t}}}_{2}\end{array}),$$

and

411 $${{\bf{v}}}_{2}\,(({\omega }_{1},{\omega }_{2};{\bf{t}}))=(\begin{array}{l}\frac{\sin \,{\omega }_{2}\,(T-{T}_{0})}{1-\,\cos \,{\omega }_{1}{T}_{0}\,\cos \,{\omega }_{2}\,(T-{T}_{0})}\,\cos \,{\omega }_{1}{{\bf{t}}}_{1}\\ \frac{\cos \,{\omega }_{1}{T}_{0}\,\sin \,{\omega }_{2}\,(T-{T}_{0})}{1-\,\cos \,{\omega }_{1}{T}_{0}\,\cos \,{\omega }_{2}\,(T-{T}_{0})}\,\cos \,{\omega }_{2}{{\bf{t}}}_{2}+\,\sin \,{\omega }_{2}{{\bf{t}}}_{2}\end{array}).$$

Using Equations (4.9–4.11), and dropping the dependencies in notation, simplifies (4.1) into

4.12 $$\mathop{minimize}\limits_{{\omega }_{1},{\omega }_{2},{b}_{1},{b}_{2},\bar{p}}\,\parallel {\bf{Q}}+\bar{p}{\bf{1}}-{\bf{f}}{\parallel }_{2}^{2}.$$

This simplification has helped to eliminate the constraints in the optimization problem (4.1).

The minimization problem (4.12) is non-convex and non-linear in its parameters. So, in order to be able to solve the problem, we can use the fact that the minimum of a function can first be found over some variables and then over the remaining ones^28^. In other words, the optimization problem in (4.12) can be written as

413 $$\mathop{minimize}\limits_{{\omega }_{1},{\omega }_{2}}\,(\mathop{minimize}\limits_{{b}_{1},{b}_{2},\bar{p}}\,\parallel {\bf{Q}}+\bar{p}{\bf{1}}-{\bf{f}}{\parallel }_{2}^{2})\mathrm{.}$$

We call the inner optimization in (4.13) as *P*(*ω*_1_, *ω*_2_). Solving for *P*(*ω*_1_, *ω*_2_) is a classical least squares problem. The solution existence and uniqueness of this optimization is mentioned in our previous work^13^. To find the exact solution we simplify the objective function as

4.14 $$\begin{array}{rcl}\parallel {\bf{Q}}+\bar{p}{\bf{1}}-{\bf{f}}{\parallel }_{2}^{2} & = & ({\bf{Q}}+\bar{p}{\bf{1}}-{\bf{f}})^{\prime} \,({\bf{Q}}+\bar{p}{\bf{1}}-{\bf{f}})\\  & = & {\bf{Q}}^{\prime} {\bf{Q}}+2\bar{p}{\bf{Q}}^{\prime} {\bf{1}}-2{\bf{Q}}^{\prime} {\bf{f}}-2\bar{p}{\bf{f}}^{\prime} {\bf{1}}+\bar{p}{\bf{1}}^{\prime} {\bf{1}}+{\bf{f}}^{\prime} {\bf{f}}.\end{array}$$

Substituting for **Q** = *b*_1_**v**_1_ + *b*_2_**v**_2_, we convert (4.14) into

4.15 $$\begin{array}{rcl}\parallel {\bf{Q}}+\bar{p}{\bf{1}}-{\bf{f}}{\parallel }_{2}^{2} & = & {b}_{1}^{2}{{\bf{v}}{\boldsymbol{^{\prime} }}}_{1}{{\bf{v}}}_{1}+2{b}_{1}{b}_{2}{{\bf{v}}{\boldsymbol{^{\prime} }}}_{1}{{\bf{v}}}_{2}+{b}_{2}^{2}{{\bf{v}}{\boldsymbol{^{\prime} }}}_{2}{{\bf{v}}}_{2}+2\bar{p}{b}_{1}{{\bf{v}}{\boldsymbol{^{\prime} }}}_{1}{\bf{1}}+2\bar{p}{b}_{2}{{\bf{v}}{\boldsymbol{^{\prime} }}}_{2}{\bf{1}}\\  &  & -\,2{b}_{1}{{\bf{v}}{\boldsymbol{^{\prime} }}}_{1}{\bf{f}}-2{b}_{2}{{\bf{v}}{\boldsymbol{^{\prime} }}}_{2}{\bf{f}}-2\bar{p}{\bf{f}}^{\prime} {\bf{1}}+{\bar{p}}^{2}{\bf{1}}^{\prime} {\bf{1}}+{\bf{f}}^{\prime} {\bf{f}}.\end{array}$$

Since, in this part of the optimization, the values of *ω*_1_ and *ω*_2_ are fixed, we can find the optimal values of *b*_1_, *b*_2_, and $\bar{p}$ by setting the partial derivatives of (4.15) equal to zero. In other words, we set $\frac{\partial (\parallel {\bf{Q}}+\bar{p}{\bf{1}}-{\bf{f}}{\parallel }_{2}^{2})}{\partial {b}_{1}}=0$, $\frac{\partial (\parallel {\bf{Q}}+\bar{p}{\bf{1}}-{\bf{f}}{\parallel }_{2}^{2})}{\partial {b}_{2}}=0$, and $\frac{\partial (\parallel {\bf{Q}}+\bar{p}{\bf{1}}-{\bf{f}}{\parallel }_{2}^{2})}{\partial \bar{p}}=0$. Doing this, we find the optimal solution for *b*_1_, *b*_2_, and $\bar{p}$, by

416 $$(\begin{array}{c}{b}_{1}^{\ast }\,({\omega }_{1},{\omega }_{2})\\ {b}_{2}^{\ast }\,({\omega }_{1},{\omega }_{2})\\ {\bar{p}}^{\ast }\,({\omega }_{1},{\omega }_{2})\end{array})={(\begin{array}{ccc}{{\bf{v}}{\boldsymbol{^{\prime} }}}_{1}{{\bf{v}}}_{1} & {{\bf{v}}{\boldsymbol{^{\prime} }}}_{1}{{\bf{v}}}_{2} & {{\bf{v}}{\boldsymbol{^{\prime} }}}_{1}{\bf{1}}\\ {{\bf{v}}{\boldsymbol{^{\prime} }}}_{1}{{\bf{v}}}_{2} & {{\bf{v}}{\boldsymbol{^{\prime} }}}_{2}{{\bf{v}}}_{2} & {{\bf{v}}{\boldsymbol{^{\prime} }}}_{2}{\bf{1}}\\ {{\bf{v}}{\boldsymbol{^{\prime} }}}_{1}{\bf{1}} & {{\bf{v}}{\boldsymbol{^{\prime} }}}_{2}{\bf{1}} & {\bf{1}}^{\prime} {\bf{1}}\end{array})}^{-1}(\begin{array}{c}{{\bf{v}}{\boldsymbol{^{\prime} }}}_{1}{\bf{f}}\\ {{\bf{v}}{\boldsymbol{^{\prime} }}}_{2}{\bf{f}}\\ {\bf{1}}^{\prime} {\bf{f}}\end{array}).$$

Here, we have fulfilled the optimization part by solving a linear system. This could potentially accelerate the IF algorithm. Finally, we only have to solve a minimization on

4.17 $$P\,({\omega }_{1},{\omega }_{2})=\parallel {\bf{Q}}({\omega }_{1},{\omega }_{2},{b}_{1}^{\ast }\,({\omega }_{1},{\omega }_{2}),{b}_{2}^{\ast }\,({\omega }_{1},{\omega }_{2});{\bf{t}})+{\bar{p}}^{\ast }\,({\omega }_{1},{\omega }_{2})\,{\bf{1}}-{\bf{f}}{\parallel }_{2}^{2},$$

which is

4.18 $$\mathop{minimize}\limits_{{\omega }_{1},{\omega }_{2}}\,P\,({\omega }_{1},{\omega }_{2})\mathrm{.}$$

We note that a property of the function *P*(*ω*_1_, *ω*_2_) is its differentiability, away from its singularities. In fact, by definition, the function $\parallel {\bf{Q}}+\bar{p}{\bf{1}}-{\bf{f}}{\parallel }_{2}^{2}$ is directionally differentiable with respect to all its variables. Hence, using the results in^29,30^, we can deduce that

419 $$P\,({\omega }_{1},{\omega }_{2})=\mathop{minimize}\limits_{{b}_{1},{b}_{2},\bar{p}}\,\parallel {\bf{Q}}+\bar{p}{\bf{1}}-{\bf{f}}{\parallel }_{2}^{2}$$

is directionally differentiable with respect to *ω*_1_ and *ω*_2_. This property can be exploited if one tries to solve (4.18) using a gradient based optimization method^31^.

### Degenerate Case (cos *ω*_1_*T*_0_ cos *ω*_2_(*T* − *T*_0_) = 1)

The solution of (4.6) can be expressed as nodes of a lattice ${\mathscr{N}}$ in *ω*_1_*ω*_2_ plane. To be more specific, we have

4.20 $${\mathscr{N}}={{\rm{\Gamma }}}_{1}\cup {{\rm{\Gamma }}}_{2},$$

where

4.21 $${\Gamma }_{1}=\{({\omega }_{1},{\omega }_{2})|{\omega }_{1}{T}_{0}=(2{k}_{1}+1)\,\pi ,{\omega }_{2}\,(T-{T}_{0})=(2{k}_{2}+1)\,\pi ,{k}_{1}\in {\mathbb{Z}},{k}_{2}\in {\mathbb{Z}}\},$$

and

4.22 $$\begin{array}{c}{\Gamma }_{2}=\{({\omega }_{1},{\omega }_{2})|{\omega }_{1}{T}_{0}=2{k}_{1}\pi ,{\omega }_{2}\,(T-{T}_{0})=2{k}_{2}\pi ,{k}_{1}\in {\mathbb{Z}},{k}_{2}\in {\mathbb{Z}}\}\mathrm{.}\end{array}$$

If (*ω*_1_, *ω*_2_) ∈ *Γ*_1_, from (4.5) we have *a*_1_ = −*a*_2_. On the other hand, if (*ω*_1_, *ω*_2_) ∈ Γ_2_, from (4.5) we have *a*_1_ = *a*_2_. In both of these cases, we can express (4.3) as

4.23 $${\bf{S}}\,({\omega }_{1},{\omega }_{1},{a}_{1},{b}_{1},{b}_{2},\bar{p};{\bf{t}})={\bf{Q}}\,({\omega }_{1},{\omega }_{1},{a}_{1},{b}_{1},{b}_{2};{\bf{t}})+\bar{p}{\bf{1}},$$

where ${\bf{Q}}({\omega }_{1},{\omega }_{1},{a}_{1},{b}_{1},{b}_{2};{\bf{t}})$  =  ${a}_{1}{{\bf{w}}}_{0}^{{{\rm{\Gamma }}}_{i}}({\omega }_{1},{\omega }_{2};{\bf{t}})+{b}_{1}{{\bf{w}}}_{1}({\omega }_{1},{\omega }_{2};{\bf{t}})+{b}_{2}{{\bf{w}}}_{2}({\omega }_{1},{\omega }_{2};{\bf{t}})$, for *i* = 1, 2. If (*ω*_1_, *ω*_2_) ∈ *Γ*_1_,

424 $${{\bf{w}}}_{0}^{{\Gamma }_{1}}=(\begin{array}{c}\begin{array}{c}\cos \,{\omega }_{1}{{\bf{t}}}_{1}\end{array}\\ -\cos \,{\omega }_{2}{{\bf{t}}}_{2}\end{array}).$$

Similarly, if (*ω*_1_, *ω*_2_) ∈ *Γ*_2_, we have

425 $${{\bf{w}}}_{0}^{{\Gamma }_{2}}=(\begin{array}{c}\begin{array}{c}\cos \,{\omega }_{1}{{\bf{t}}}_{1}\end{array}\\ \cos \,{\omega }_{2}{{\bf{t}}}_{2}\end{array})\mathrm{.}$$

In both of the cases, we have

426 $${{\bf{w}}}_{1}=(\begin{array}{c}\sin \,{\omega }_{1}{{\bf{t}}}_{1}\\ {{\bf{0}}}_{1}\end{array}),$$

and

427 $${{\bf{w}}}_{2}=(\begin{array}{c}{{\bf{0}}}_{2}\\ \sin \,{\omega }_{2}{{\bf{t}}}_{2}\end{array})\mathrm{.}$$

Here, **0**_1_ and **0**_2_ are zero vectors in ${{\mathbb{R}}}^{m\times 1}$ and ${{\mathbb{R}}}^{n\times 1}$, respectively. It is clear, from (4.26) and (4.27), that ${{\bf{w}}{\boldsymbol{^{\prime} }}}_{1}{{\bf{w}}}_{2}={{\bf{w}}{\boldsymbol{^{\prime} }}}_{2}{{\bf{w}}}_{1}=0$. Using (4.23), and a similar approach we employed in (4.15) and (4.16), we find the optimal solution for *a*_1_, *b*_1_, *b*_2_, and $\bar{p}$, by

428 $$(\begin{array}{c}{a}_{1,i}^{\ast }({\omega }_{1},{\omega }_{2})\,\\ {b}_{1,i}^{\ast }\,({\omega }_{1},{\omega }_{2})\\ {b}_{2,i}^{\ast }\,({\omega }_{1},{\omega }_{2})\\ {\bar{p}}_{i}^{\ast }\,({\omega }_{1},{\omega }_{2})\end{array})={(\begin{array}{cccc}({{\bf{w}}}_{0}^{{\Gamma }_{i}})^{\prime} {{\bf{w}}}_{0}^{{\Gamma }_{i}} & ({{\bf{w}}}_{0}^{{\Gamma }_{i}})^{\prime} {{\bf{w}}}_{1} & ({{\bf{w}}}_{0}^{{\Gamma }_{i}})^{\prime} {{\bf{w}}}_{2} & ({{\bf{w}}}_{0}^{{\Gamma }_{i}})^{\prime} {\bf{1}}\\ ({{\bf{w}}}_{0}^{{\Gamma }_{i}})^{\prime} {{\bf{w}}}_{1} & {{\bf{w}}{\boldsymbol{^{\prime} }}}_{1}{{\bf{w}}}_{1} & 0 & {{\bf{w}}{\boldsymbol{^{\prime} }}}_{1}{\bf{1}}\\ ({{\bf{w}}}_{0}^{{\Gamma }_{i}})^{\prime} {{\bf{w}}}_{2} & 0 & {{\bf{w}}{\boldsymbol{^{\prime} }}}_{2}{{\bf{w}}}_{2} & {{\bf{w}}{\boldsymbol{^{\prime} }}}_{2}{\bf{1}}\\ ({{\bf{w}}}_{0}^{{\Gamma }_{i}})^{\prime} {\bf{1}} & {{\bf{w}}{\boldsymbol{^{\prime} }}}_{1}{\bf{1}} & {{\bf{w}}{\boldsymbol{^{\prime} }}}_{2}{\bf{1}} & {\bf{1}}^{\prime} {\bf{1}}\end{array})}^{-1}(\begin{array}{c}({{\bf{w}}}_{0}^{{\Gamma }_{i}})^{\prime} {\bf{f}}\\ {{\bf{w}}{\boldsymbol{^{\prime} }}}_{1}\,{\bf{f}}\\ {{\bf{w}}{\boldsymbol{^{\prime} }}}_{2}\,{\bf{f}}\\ {\bf{1}}^{\prime} \,{\bf{f}}\end{array}),$$

for *i* = 1, 2. Hence, similar to (4.17), for (*ω*_1_, *ω*_2_) ∈ Γ_1_ or (*ω*_1_, *ω*_2_) ∈ Γ_2_, we only have to solve a minimization on

4.29 $$P\,({\omega }_{1},{\omega }_{2})=\parallel {\bf{Q}}\,({\omega }_{1},{\omega }_{1},{a}_{\mathrm{1,}i}^{\ast }({\omega }_{1},{\omega }_{2}),{b}_{\mathrm{1,}i}^{\ast }\,({\omega }_{1},{\omega }_{2}),{b}_{\mathrm{2,}i}^{\ast }\,({\omega }_{1},{\omega }_{2});{\bf{t}})+{\bar{p}}_{i}^{\ast }\,({\omega }_{1},{\omega }_{2}){\bf{1}}-{\bf{f}}{\parallel }_{2}^{2}\mathrm{.}$$

Note that, from a machine learning perspective, the nodes specified in (4.20) do not have important information physiologically as they could be inferred from the systolic and diastolic parts of a waveform alone. In other words, even if these points present a global minima, they are not informative as we already know the systolic and diastolic inverses, $\frac{1}{{T}_{0}}$ and $\frac{1}{T-{T}_{0}}$ respectively, as possible inputs to any machine learning algorithm. Hence, these points could possibly be ignored in a search for an optimum point of (4.1).

## Fast IF Algorithms

In this section, we present a fast IF algorithm which is based on the results presented in the previous section and the topology of the solution space for *P*(*ω*_1_, *ω*_2_). In order to keep the fluency of this section, we mention the original IF algorithm (see Algorithm 1) as presented in^13^.

Algorithm 1 has three major steps. In the first step, the (*ω*_1_,*ω*_2_) domain

5.1 $${{\mathscr{D}}}_{fr}=\{({\omega }_{1},{\omega }_{2})|0 < {\omega }_{1}\le C,0 < {\omega }_{2}\le C\}$$

is made discrete, namely ${\overline{{\mathscr{D}}}}_{fr}$. The second step is a minimization to find *P*(*ω*_1_, *ω*_2_), see (4.18). The final step is a brute-force search on ${\overline{{\mathscr{D}}}}_{fr}$ to find the minimum of *P*(*ω*_1_, *ω*_2_).

All three steps can be optimized to make the IF algorithm faster. Regarding the domain of optimization ${{\mathscr{D}}}_{fr}$, defined in (5.1), we know from our previous work in^14^ that the average IF solution, for a physiological pulse waveform recording, is confined to a smaller domain ${\mathscr{D}}$ expressed as

5.2 $${\mathscr{D}}=\{({\omega }_{1},{\omega }_{2})|0.5\leqslant \tfrac{{\omega }_{1}{T}_{0}}{\pi }\leqslant 1.5,0.5\leqslant \tfrac{{\omega }_{2}(T-{T}_{0})}{\pi }\leqslant 3\}.$$

This will make the first step search area more well-defined and optimized. In the previous section, we have been able to find some analytic solutions (see (4.16)) for the inner optimization part of problem (4.13). This will help us to substitute an analytic solution instead of an iterative^32^ or QR decomposition^33^ solution for (5.3). Finally, the brute-force part can be substituted with an appropriate direct search algorithm^34^, e.g. pattern search algorithm^35^. It can even be substituted with an appropriate gradient based algorithm^28,31^, e.g. gradient descent, as we know the differentiability of *P*(*ω*_1_, *ω*_2_).

**Algorithm 1:**
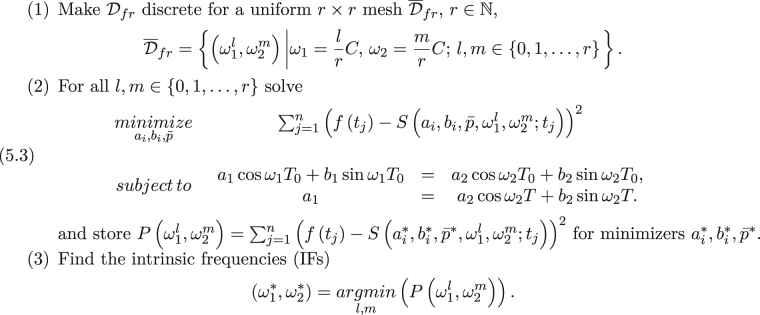
Intrinsic Frequency.

**Figure 2:**
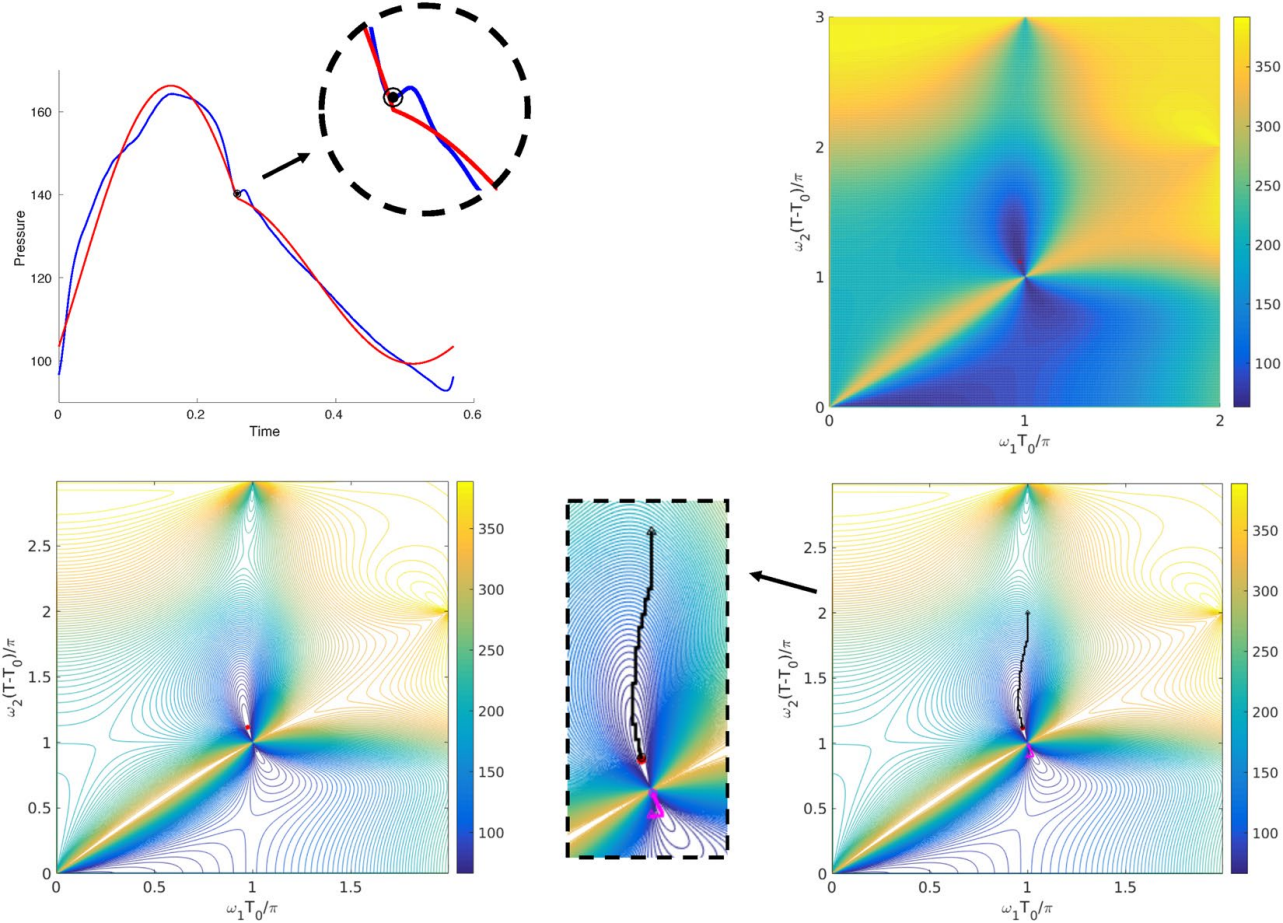
Up-Left: A dog aortic pressure cycle (in blue), its dicrotic notch (black circle with dotted center), and the IMF (in red). Up-Right: heat-map plot of $P(\frac{{\omega }_{1}{T}_{0}}{\pi },\frac{{\omega }_{2}(T-{T}_{0})}{\pi })$ for the cycle in left with the location of the solution marked with red dot. Down-Left: Contour plot of $P(\frac{{\omega }_{1}{T}_{0}}{\pi },\frac{{\omega }_{2}(T-{T}_{0})}{\pi })$. The location of the minimizer of *P* is shown by a red dot. Down-Right: Contour plot of $P(\frac{{\omega }_{1}{T}_{0}}{\pi },\frac{{\omega }_{2}(T-{T}_{0})}{\pi })$ and the location of the minimizer of *P* tracked by the pattern search. The beginning of the pattern search is marked with a triangle and its end with a circle. The true optimum point is marked with a red dot. The upper pattern search set (in black) has converged towards the correct optimum. The lower pattern search set (in magenta) has converged to a local minima near the node.

**Figure 3:**
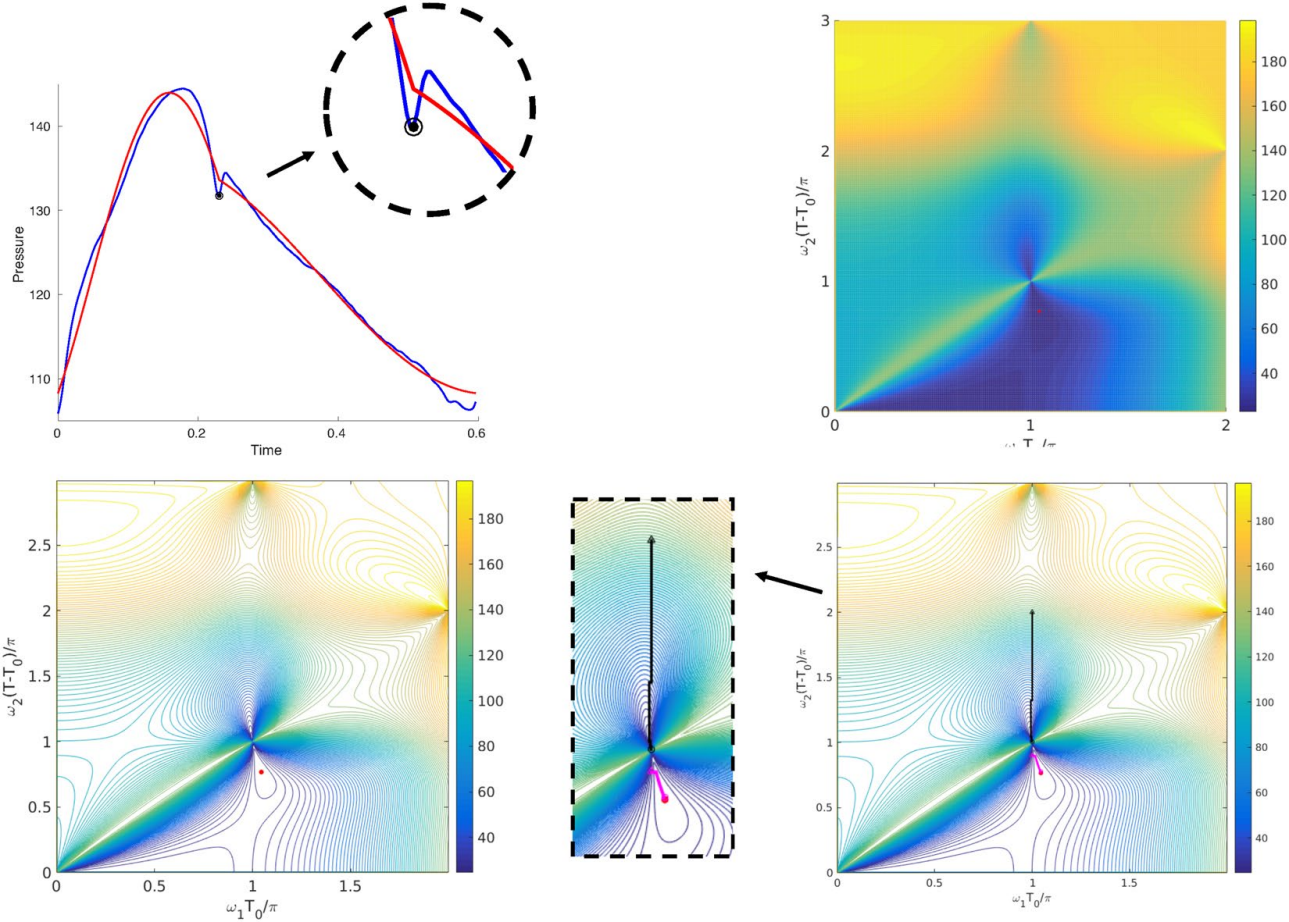
Up-Left: A dog aortic pressure cycle (in blue), its dicrotic notch (black circle with dotted center), and the IMF (in red). Up-Right: heat-map plot of $P(\frac{{\omega }_{1}{T}_{0}}{\pi },\frac{{\omega }_{2}(T-{T}_{0})}{\pi })$ for the cycle in left with the location of the solution marked with red dot. Down-Left: Contour plot of $P(\frac{{\omega }_{1}{T}_{0}}{\pi },\frac{{\omega }_{2}(T-{T}_{0})}{\pi })$. The location of the minimizer of *P* is shown by a red dot. Down-Right: Contour plot of $P(\frac{{\omega }_{1}{T}_{0}}{\pi },\frac{{\omega }_{2}(T-{T}_{0})}{\pi })$ and the location of the minimizer of *P* tracked by the pattern search. The beginning of the pattern search is marked with a triangle and its end with a circle. The true optimum point is marked with a red dot. The lower pattern search set (in black) has converged towards the correct optimum. The upper pattern search set (in magenta) has converged to a local minima near the node.

Before moving on, we show the topology of the *P*(*ω*_1_, *ω*_2_) function and also its minima locations in *ω*_1_ and *ω*_2_ space. These will provide useful insights on where to set the initialization point(s) of a possible fast IF algorithm. The data description is provided in the next section. In Figs 2 and 3, we have presented two different dog aortic pressure cycles with the IMF extracted by the means of the brute-force IF Algorithm 1. Figures 2 and 3, top right, show the heat-map plots of $P(\frac{{\omega }_{1}{T}_{0}}{\pi },\frac{{\omega }_{2}(T-{T}_{0})}{\pi })$. The complex nature of *P*(*ω*_1_, *ω*_2_) can be seen in these figures. We purposefully plotted *P* in the dimensionless coordinates $\frac{{\omega }_{1}{T}_{0}}{\pi }$ and $\frac{{\omega }_{2}(T-{T}_{0})}{\pi }$ to show the behavior of this function with respect to the lattice node locations ${\mathscr{N}}$ defined in (4.20–4.22). To have a better view and understanding of the *P*(*ω*_1_,*ω*_2_) topology, a contour of $P(\frac{{\omega }_{1}{T}_{0}}{\pi },\frac{{\omega }_{2}(T-{T}_{0})}{\pi })$ is shown in those figures. The general topology of $P(\frac{{\omega }_{1}{T}_{0}}{\pi },\frac{{\omega }_{2}(T-{T}_{0})}{\pi })$, for all aortic or carotid pulse waveforms, is similar to the ones presented in Figs 2 and 3. However, the location of the minimizer is not similar.

Our investigations show that the locations of the minimizers of all *P* functions construct two different areas in the dimensionless coordinates $\frac{{\omega }_{1}{T}_{0}}{\pi }$ and $\frac{{\omega }_{2}(T-{T}_{0})}{\pi }$. We call these areas as the *upper lobe* and *lower lobe*. The upper lobe is an area, in ${\mathscr{D}}$, confined above the line $\frac{{\omega }_{2}(T-{T}_{0})}{\pi }=1$. The lower lobe is an area, in ${\mathscr{D}}$, confined below the line $\frac{{\omega }_{2}(T-{T}_{0})}{\pi }=1$. This is also the case for human subject data^14^. This type of topology suggests two critical initial guess areas for any non-brute-force algorithm solving (4.1): one set of points in the upper lobe, the other in the lower. In the remaining part of this section, we introduce a fast IF algorithm based on the pattern search method^34^.

### Pattern Search IF

The pattern search algorithm (or sometimes called the *compass search* algorithm) is explained in detail in^34^. For completeness, we have summarized the pattern search algorithm in Algorithm 2. The convergence analysis of this method is expressed in^34^.

**Algorithm 2:**
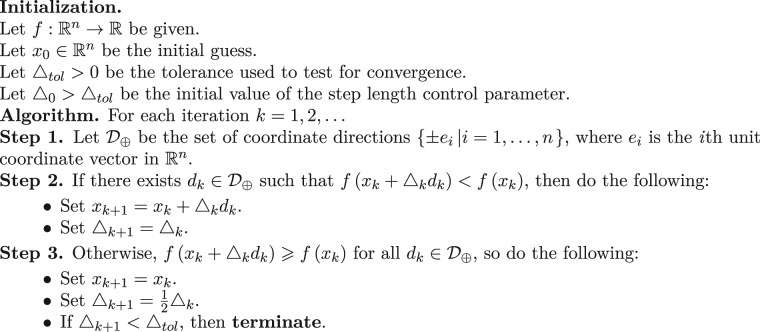
Pattern Search^34^.

The fast IF algorithm, without considering the nodes (4.20), is expressed in Algorithm 3. As mentioned before, what makes Algorithm 3 fast is embedded in three different objects:

1. The initial guess set up in the initialization part of the algorithm.
2. The fast analytic solution at each point iteration defined by (4.16) and (4.17).
3. The pattern search part which is a substitute for the brute force algorithm.

Figure 2, bottom right, shows the results of Algorithm 3. In this figure, when using Algorithm 3, we have used two initial guesses $(\frac{{\omega }_{1}{T}_{0}}{\pi }=1,\frac{{\omega }_{2}(T-{T}_{0})}{\pi }=2)$ and $(\frac{{\omega }_{1}{T}_{0}}{\pi }=1,\frac{{\omega }_{2}(T-{T}_{0})}{\pi }=0.9)$. As depicted on the figure, the initial guess located in the upper lobe has converged towards the true minimizer in ${\mathscr{D}}$. On a PC having 8 threads, Intel® Core™ i7-4700MQ CPU @ 2.40 GHz × 8, running a Matlab implementation of the brute-force Algorithm 1 in parallel takes roughly 85 seconds. On the other hand, achieving the same minimizer, using a sequential version of the fast Algorithm 3, takes approximately 0.5 seconds. Comparing the brute-force and pattern search algorithms, the absolute error in estimating *ω*_1_ is ~0.02 (an absolute error of ~0.002 in $\tfrac{{\omega }_{1}{T}_{0}}{\pi }$) and the relative error is 0.17%. The absolute error in estimating *ω*_2_ is ~0.08 (an absolute error of ~0.008 in $\tfrac{{\omega }_{2}(T-{T}_{0})}{\pi }$) and the relative error is 0.74%.

The same test was done for another aortic cycle presented in Fig. 3. We used the same initial guesses as before. This time, on the same PC, using the same implementations, the brute-force Algorithm 1 took roughly 80 seconds and the fast Algorithm 3 took approximately 0.5 seconds. These two examples show a speed up of almost 160 times. In the next section we present more about the statistical accuracy of Algorithm 3 and its physiological capabilities. Comparing the brute-force and pattern search algorithms, the absolute error in estimating *ω*_1_ is ~0.02 (an absolute error of ~0.001 in $\tfrac{{\omega }_{1}{T}_{0}}{\pi }$) and the relative error is 0.11%. The absolute error in estimating *ω*_2_ is ~0.05 (an absolute error of ~0.006 in $\tfrac{{\omega }_{2}(T-{T}_{0})}{\pi }$) and the relative error is 0.83%.

**Algorithm 3:**
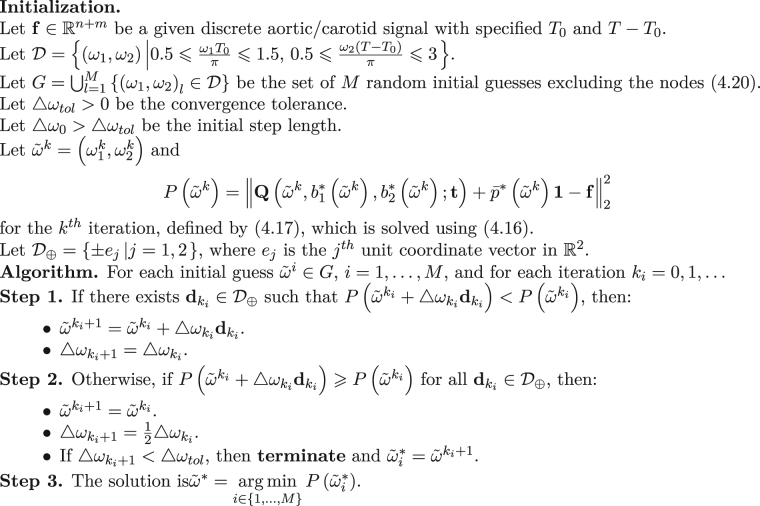
Fast IF.

## Real Data Example

### Data Description

The real dog data used in this manuscript is well described in^36^. Briefly, six normal adult beagle dogs had undergone the data collection experiment. One dog was involved in a sterile surgical procedure for implanting chronic recording transducers. All the dogs had undergone general anesthesia with propofol and maintained it with inhaled isoflorane. For the aortic pressure waveform measurements, a micro-manometer-tipped catheter was inserted into a femoral artery and guided into the descending thoracic aorta. The transducer outputs were transfered to a personal computer through an A/D conversion system. The measurements were collected over a period of 50– 170 minutes. Some pharmacological influences and total intra-vascular volume changes were imposed on the dogs: dobutamine, esmolol, verapamil, phenylephrine, nitroprusside, saline and progressive hemorrhage. For a detailed description, see^36^.

Since, at the time of the the data retrieval, the data was downloaded with different sampling rates, we re-sampled all six dog data at 500 *Hz*. We used a modified version of the automatic cycle selection introduced in^37^ to pick cycles. Dicrotic notch locations were then found from the picked cycles^38^. We totally extracted 59384 acceptable aortic cycles form those six dogs.

### Statistical Accuracy

To check the statistical accuracy of the fast IF algorithm versus the brute-force IF algorithm, we compared the results of these two algorithms on the extracted 59384 dog aortic cycles. The brute-force IF algorithm (Algorithm 1) was run over the sample set with a mesh size $(\mathop{{\rm{\min }}}\limits_{l\ne m}\,({\omega }_{1}^{l}-{\omega }_{1}^{m})=\mathop{{\rm{\min }}}\limits_{l\ne m}\,({\omega }_{2}^{l}-{\omega }_{2}^{m}))$ of 0.02*π*. Algorithm 3 was run on the same sample set of 59384 aortic cycles with Δ*ω*_*tol*_ = 0.001, and Δ*ω*_0_ = 0.1, comprising a mesh size of $\frac{0.1}{{2}^{6}}$. The brute-force algorithm has a larger mesh size due to heavy computational cost of this algorithm. The maximum average difference between the IFs found by these two algorithms was found to be less than 0.0475. This difference is smaller than both mesh sizes used for the brute-force and fast IF algorithms. This shows that, on average, the fast IF algorithm (Algorithm 3) reaches the same minima as the brute-force algorithm (Algorithm 1).

### Physiological Observations

**Figure 4:**
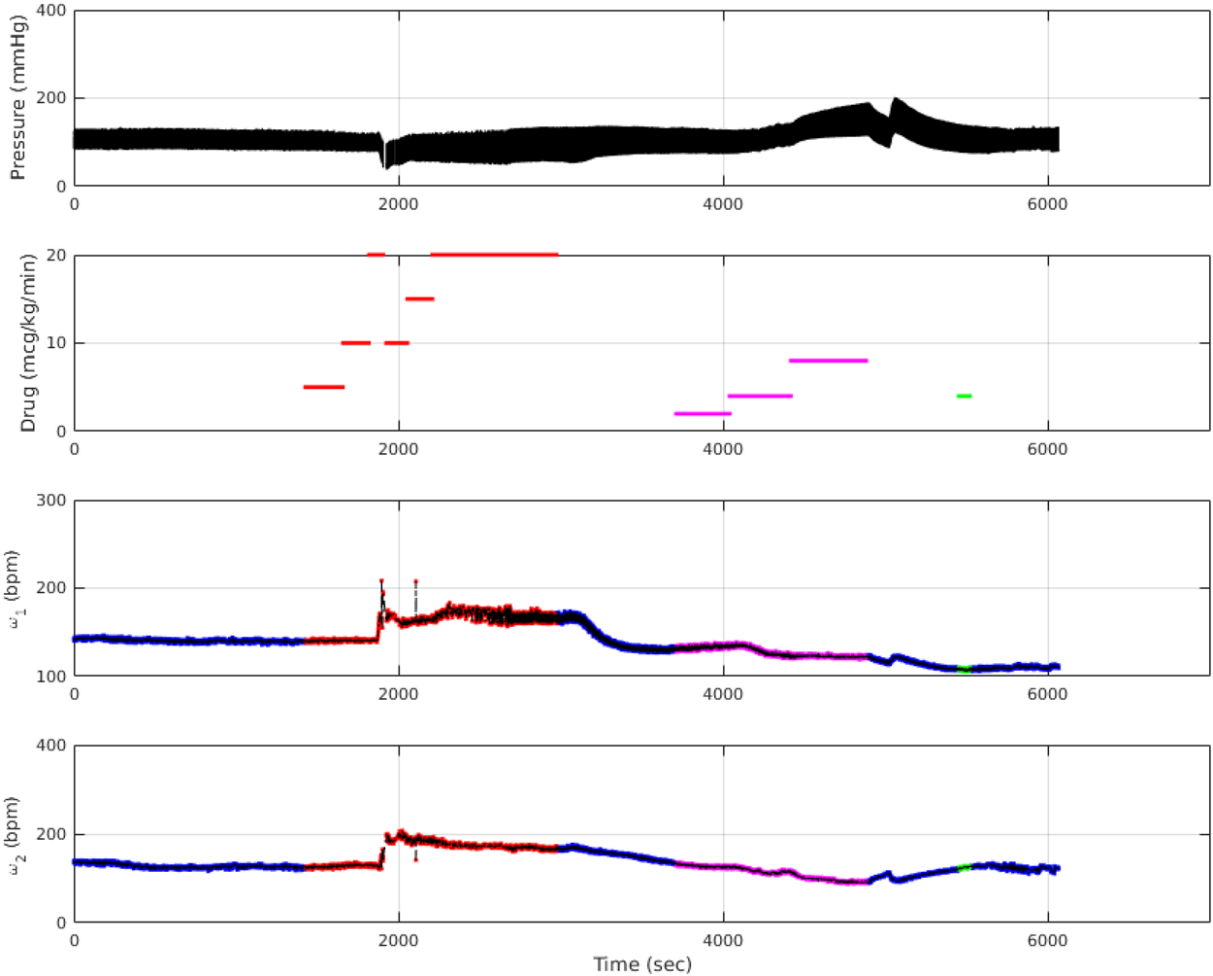
Drug effects on *ω*_1_ and *ω*_2_. First Panel: The measured aortic pressure waveform recorded in time. Second Panel: Dosage of dobutamine (in red), phenylephrine (in purple), and nitroglycerin (in green) during the aortic pressure measurement. Third Panel: Changes of *ω*_1_ in units of bit per minute (bpm) over the measurement time. Each drug effect is projected with its corresponding color, for the fast IF algorithm. No drug areas are in blue, for the fast IF algorithm. The brute-force algorithm results are shown in dashed black line. Fourth Panel: Changes of *ω*_2_ in units of bpm over the measurement time. Each drug effect is projected with its corresponding color, for the fast IF algorithm. No drug areas are in blue, for the fast IF algorithm. The brute-force algorithm results are shown in dashed black line.

To evaluate the new fast IF algorithm (Algorithm 3), we applied the algorithm on the measured aortic pressure signal from one dog experiencing various pharmacological interventions, see Fig. 4. During the experiment, the dog was under the following pharmacological influences: infusion of dobutamine (5–20 *μg*/*kg*/*min*), phenylephrine (2–8 *μg*/*kg*/*min*) and nitroglycerin (4 *μg*/*kg*/*min*) during different time intervals.

The second panel, in Fig. 4, shows the dosage and duration of each drug in the experiment. In the first phase of the experiment dobutamine has been injected at a low dosage followed by a fluctuation in the dosage of injection.

The third and fourth panels, in Fig. 4, show the trends of *ω*_1_ and *ω*_2_ for both the fast IF algorithm (Algorithm 3 in blue, red, purple and green dots) and the brute-force algorithm (Algorithm 1 in dashed black line). Both algorithms follow the same trends showing the same precision. In other words, if the force-brute IF is used as a control, the fast IF is exactly duplicating the trend.

The effect of dobutamine on the cardiovascular system is to increase the strength and force of the heartbeat. Consequently, it forces more blood to circulate throughout the body. In previous works^12,14^, we hypothesized that *ω*_1_ would be a representative of heart functionality. We also hypothesized that *ω*_1_ and *ω*_2_ would try to keep a balance during changes. These hypotheses can be seen during the injection of dobutamine in this figure.

Next, phenylephrine has been injected at a low dosage and the dosage is then increased over time. Phenylephrine is a decongestant which affects the cardiovascular system by shrinking blood vessels. *ω*_2_ shows an almost monotone decrease during the infusion of phenylephrine. This is again in qualitative accord with what we presented in^12^.

Lastly, nitroglycerin has been injected at a constant dosage. Nitroglycerin helps to dilate the blood vessels. This dilation can be captured with *ω*_2_, as can be seen from the figure. Generally, based on this figure, IFs are able to capture changes in the dynamics of the system under the effects of different drugs.

## Conclusion

In this paper, we provided a mathematical foundation for the IF model^13^. We showed how to derive an estimation of the IF model (2.1) by considering basic physics principles. More precisely, we showed that the IF model can be estimated from Navier-Stokes and elasticity equations.

We further analysed the IF model (4.1). This helped to introduce a fast algorithm for the IF method (Algorithm 3). What made this algorithm fast was embedded in the proper set up of the initial guesses based on the topology of the problem, fast analytic solution at each point iteration, and substituting the brute force algorithm with a pattern search method. These changes would convert an iterative and brute-force method (Algorithm 1) into an algebraic and iterative method (Algorithm 3). The presented fast algorithm, in this article, has a speed up of more than 100 times compared to the brute-force algorithm provided in^13^. From a statistical perspective, we have also shown that the algorithm presented in this article complies well with the brute-force implementations of this method.

We also showed, on a real dataset, that the fast IF Algorithm 3 can depict correlations between its outputs and infusion of certain drugs. This part of our paper can be subject to further physiological and clinical investigations in a future work.

### Data availability

All data generated or analysed during this study are included in the Supplementary Information files. The datasets generated during and analysed during the current study are also available from the corresponding author on reasonable request.

### Research ethics

All experiments and procedures were reviewed and approved by the MSU All-University Committee on Animal Use and Care^36^.
